# Audience presence influences cognitive task performance in chimpanzees

**DOI:** 10.1016/j.isci.2024.111191

**Published:** 2024-11-08

**Authors:** Christen Lin, Akiho Muramatsu, Shinya Yamamoto

**Affiliations:** 1 Wildlife Research Center, Kyoto University, Kyoto 606-8203, Japan; 2 Primate Research Institute, Kyoto University, Kyoto 484-8506, Japan; 3 Institute for Advanced Study, Kyoto University, Kyoto 606-8501, Japan; 4 Research and Education Center for Comprehensive Science, Akita Prefectural University, Akita 010-0195, Japan

**Keywords:** Neuroscience, Behavioral neuroscience, Cognitive neuroscience

## Abstract

Human cognitive performance can be significantly influenced by the presence of audience members. While often associated with reputation management, which is considered uniquely human, it is unclear to what degree this phenomenon is shared with non-human animals. To investigate such audience effects in chimpanzees, we recorded the performance of six chimpanzees on three different numerical touch screen tasks varying in difficulty and cognitive demand, in the presence of variable audience member compositions over six years. Our results indicated that chimpanzee performance was influenced by the number and types of audience present. Performance increased for the most difficult task as the experimenter count increased, while for the easiest task, performance decreased as familiar audience and experimenter count increased. This suggests that audience effects on cognitive processing can be found in chimpanzees and that the evolutionary roots of this trait may date back to before the development of reputation-based normative societies in humans.

## Highlights

• Six chimpanzees participated in computer-controlled cognitive tasks over six years
• Chimpanzee performance changed with the number and types of audience
• Audiences may influence the perception of reward, cognitive load, and concentration
• Audience effects may have emerged before the development of reputation-based society

## Graphical abstract

**Figure:**
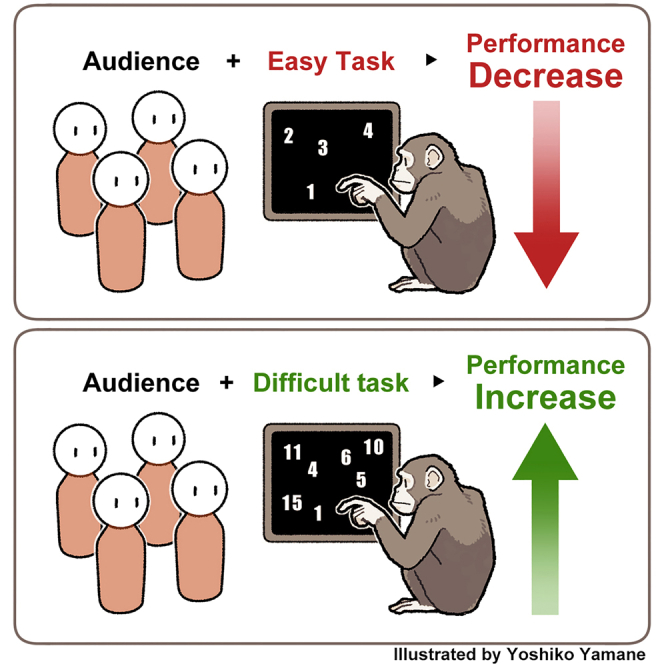

## Introduction

The uniqueness of human cognition and sociality may largely be in part due to the special notice we give toward how others view us. Even just the perceived presence of a potential witness can affect human cognitive performance and decision-making.^1^^,^^2^ It is evident that humans place a great deal of importance on the judgment of their peers; many other studies have found that humans even tend to alter their decision-making and behavior when they believe they are being watched.^2^^,^^3^^,^^4^^,^^5^ This can often be observed as the watching-eye effect, which is a phenomenon describing the tendency of people to act as though they are being watched when some form of eyes are present (even rudimentary or graphical representations of them).^1^ Other phenomena describing how the perception of an audience can influence one’s performance include social inhibition and facilitation.^6^^,^^7^ Several studies have found that for difficult tasks, such as those involving memory or learning, people experienced a decrease in their performance while being watched by a third party.^8^^,^^9^^,^^10^^,^^11^ It is possible that performing a task in the presence of other people results in an increased pressure to perform well,^12^ and for tasks that are more cognitively complex, it may be the case that the sum of this increased social pressure along with the difficulty of the task leads to poorer performance overall. On the other hand, the presence of witnesses can also enhance performance through social facilitation. Previous studies have found that in the presence of others, subjects were able to better navigate a maze^12^ and performed better on a simple typing task while being observed, although performance for a more difficult task decreased in the presence of witnesses.^13^

The significance of witnesses to humans under various contexts could be explained by the importance of reputation management to our species. Social norms and indirect reciprocity are two key aspects of human society that contribute to the prominence of reputation management^14^^,^^15^; those who tend to act in a socially acceptable manner are evaluated as such by their peers, and in turn their future social interactions may benefit if witnesses spread the word of their good deeds. Of course, those who are witnessed violating societal norms would also eventually be punished through the same means.^16^^,^^17^^,^^18^ With how vital reputation management is due to these social mechanisms, it would also be highly beneficial to pay special attention to the witnesses of one’s actions in order to maximize potential benefits to one’s social standing. Thus, traits that allowed humans to alter their behavior based on the types of witnesses involved would have improved fitness through improving their reputations among their peers.

While these qualities are not necessarily unique to humans, they are certainly special in terms of how humans utilize them.^19^ Even so, cross-species comparisons of such characteristics may potentially shed more light on the origins of such features in humans; in particular, examining whether similar traits are present in our great ape relatives might help reveal whether these features arose before or after our divergence.^20^ Many other non-human animals also exhibit broad behavioral changes in the presence of conspecifics. Such alterations of behavior in response to others being present are referred to as audience effects, which have been observed across a wide range of species.^21^ For instance, Thomas langurs only cease their predator warning calls once they have confirmed a call-in response from all other group members.^21^^,^^22^ Studies on captive zoo animals have examined changes in primate behavior based on the number of human audiences as well; Japanese macaques were less accurate in a match-to-sample task as human audience count increased,^23^ and gorillas were also negatively impacted by zoo visitors.^24^ In chimpanzees, one of our closest evolutionary relatives, results have been mixed. Previous studies have examined how chimpanzees change their gestures and other methods of communication based on the characteristics of conspecific audiences,^25^^,^^26^ and they have been found to change their food calls based on the rank of their audience.^27^ Other studies related to social learning have also found that chimpanzees appear to be better at emulative learning in the presence of conspecifics.^28^^,^^29^^,^^30^ However, another study found that chimpanzee engagement with a foraging task was not impacted by an increase in human audiences^31^ (for review on zoo visitor impacts, see the study by Sherwen and Hemsworth^32^). Furthermore, a previous study that directly tested for the watching-eye effect in chimpanzees by seeing if the presence of chimpanzee images with prominent eyes affected their choice to take food found that, overall, chimpanzees were apparently not influenced by watching eyes, or at least not to the extent that humans are.^33^

Therefore, the evolutionary roots of audience effects in the great ape lineage are still not fully unveiled, and the number of studies investigating how audiences impact chimpanzee performance and cognition on complex tasks is still lacking. In our study, we aimed to examine the presence of potential audience effects on cognitive task performance in chimpanzees using human audience members instead, as we worked with a unique group of chimpanzees who are very accustomed to human interaction. Using computer task performance data collected over the past several years with six chimpanzees, we sought to analyze whether or not the chimpanzees’ performance was influenced by the presence, quantity, and type of human audience members. If chimpanzees do not demonstrate any audience effect on their cognitive performance, we expect that chimpanzee task performance here would not be affected by the number and types of human audience members present since the presence of audience is irrelevant to the task itself.

## Results

### Performance across task types

We analyzed performance data on three different types of computer-controlled cognitive tasks from six chimpanzees residing at the Primate Research Institute (PRI, recently re-organized into the Center for the Evolutionary Origins of Human Behavior: EHUB) of Kyoto University, over a six-year period from 2009 to 2015. Three task types were utilized in our final analysis, referred to as task types 1, 2, and 3. These tasks used Arabic numerals as stimuli, requiring participants to touch the numerals in ascending order. In task type 1, numbers from a range of 1–19 would appear sequentially, while for task type 2, the numbers were not directly adjacent but were still in ascending order. Task type 3 was similar to type 2, except after the initial number was pressed, the other numbers would be hidden, requiring rapid memorization of their positions (refer to the data collection and procedure section under methods for more detailed explanations on the task types).^34^^,^^35^^,^^36^ To examine the differences in performance between the three task types, we performed a one-way ANOVA comparing task accuracy versus task type. There was a statistically significant difference in performance between the task types (*p* < 0.001; Table S4), and subsequent analysis of this model indicated a significant difference in performance between both task types 1 and 2 with task type 3 (*p* < 0.001 and *p* < 0.001, respectively; Table S5), suggesting that the task types varied in difficulty, with the lowest mean performance for task type 3 (task type 1 vs. task type 3 mean difference, 95% confidence interval [CI]: lower = −17.946, upper = −16.066; task type 2 vs. task type 3 mean difference, 95% CI: lower = −17.674, upper = −16.067; see Tables S2 and S3 for more detailed results).

### Model 1: Performance does not change with the presence of extra audience members

After confirming a difference in performance across task types, we modeled the data with various generalized linear mixed models to examine the effect of audience on performance. The first model aimed to assess if the general presence of audience members, which included familiar and unfamiliar audience members but excluded experimenters, corresponded to a change in performance (refer to Table S2 for a more detailed explanation of the different audience types). Familiar audience members were those the chimpanzees had seen before, while unfamiliar members were people the chimpanzees had not seen previously. Experimenters would manage the touch screen and the automatic feeder during the experiment, while familiar audiences were also recognizable to the chimpanzees but were more passive audience members who simply watched. In this first model, the interaction between the presence of audience members and task type was a fixed effect along with task serial number (to account for time variance in performance), while subject and task ID were the random effects. The first model included all three task types together, and the results of the likelihood-ratio test indicated that the interaction effect between the presence of audience and task type was statistically significant (d.f. = 9, χ^2^ = 9.870, *p* = 0.007; Table S6). Following this, to examine the effects of presence within each task type, each one was modeled separately. For all task types, the fixed effect of audience presence was predicted to be statistically non-significant to the model (task type 1: *p* = 0.112, task type 2: *p* = 0.917, task type 3: *p* = 0.338; Table S6). More details on the models used, their corresponding formulas, and 95% CI values can be found in Table S6.

### Model 2: Performance changes with the number of total audience present

**Figure 1:**
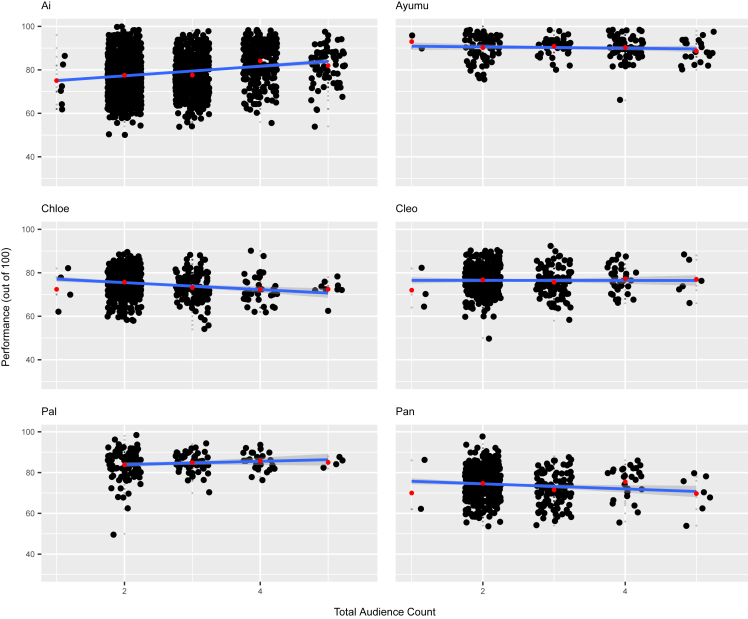
Chimpanzee performance at different total audience counts for task type 1 (easiest task) Scatterplots of performance out of 100% on task type 1 on the y axes, plotted against total audience count on the x axes. Plots are separated by individual, and subject names are listed in the top left corner of each plot. Included on each plot is an estimated linear model represented by a blue line, with the gray area representing the 95% confidence interval of the linear prediction. Red points indicate the average performance value for the corresponding column’s total audience count. For the sake of clarity, horizontal and vertical jitter were applied to the data points. Each small gray point represents the performance for one experimental session. Larger black points represent exact performance values that were reached in more than one session. Sample sizes per individual were as follows, in order from left to right, top to bottom: *n* = 2182, 206, 575, 415, 179, and 598. See also Table S7.

After examining the effects of general audience presence on performance, our next goal was to more closely investigate the relationship between the quantity of audience members and task performance. In model 2, the interaction between total audience number and task type, along with task serial number, was included as the fixed effects. The likelihood-ratio test for the initial model indicated that the interaction between total audience count and task type was statistically significant (d.f. = 9, χ^2^ = 40.170, *p* < 0.001; Table S7). Follow-up models for each task type were constructed to investigate the specific relationship between each task type and performance. In the model for task type 1, the total audience count fixed effect had a statistically significant negative relationship with performance. For this model, per unit increase in total audience number, performance in task type 1 was predicted to decrease by approximately 3%–6% (LRT: d.f. = 5, χ^2^ = 27.931, *p* < 0.001; 95% CI odds ratio: 0.938, 0.971; Figure 1, or see Table S7 for further details).

### Model 3: Performance changes with the number of experimenters and familiar audience

In our final model, our goal was to examine the effects of each specific audience type count. In model 3, the interaction between the corresponding quantities for each specific audience type (experimenter, familiar audience, and unfamiliar audience) and task type was included as separate fixed effects, along with task serial number. In the initial model including all task types, the interaction between experimenter count and task type was significant (d.f. = 9, χ^2^ = 45.270, *p* < 0.001), as well as the interaction between familiar audience count and task type (d.f. = 9, χ^2^ = 30.920, *p* < 0.001; Table S8).

**Figure 2:**
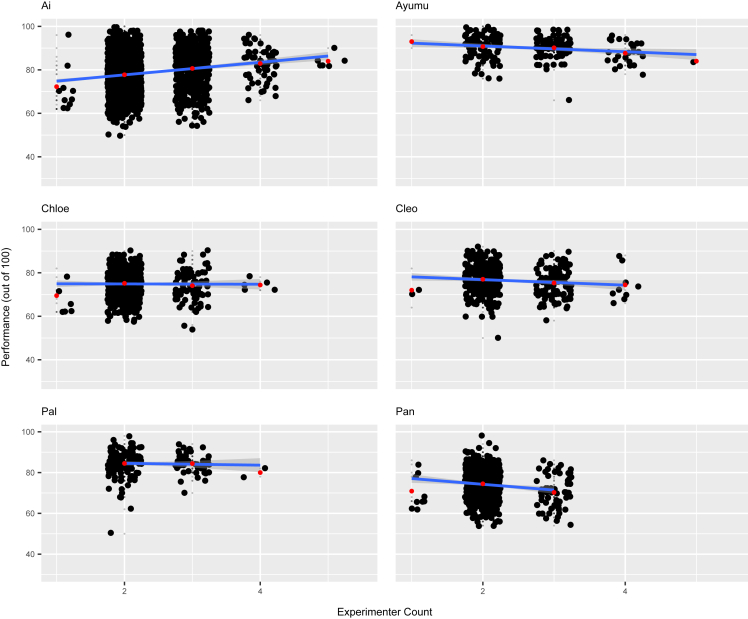
Chimpanzee performance at different experimenter audience counts for task type 1 (easiest task) Scatterplots of performance out of 100% on task type 1 against experimenter counts. Plots are separated by individual, and subject names are listed in the top left corner of each plot. Included on each plot is an estimated linear model represented by a blue line, with the gray area representing a 95% confidence interval of the linear prediction. Red points indicate the average performance value for the given experimenter count of that column. For the sake of clarity, horizontal and vertical jitter were applied to the data points. Each small gray point represents the performance for one experimental session. Larger black points represent exact performance values that were reached in more than one session. Sample sizes per individual were as follows, in order from left to right, top to bottom: *n* = 2182, 206, 575, 415, 179, and 598. See also Table S8.

**Figure 3:**
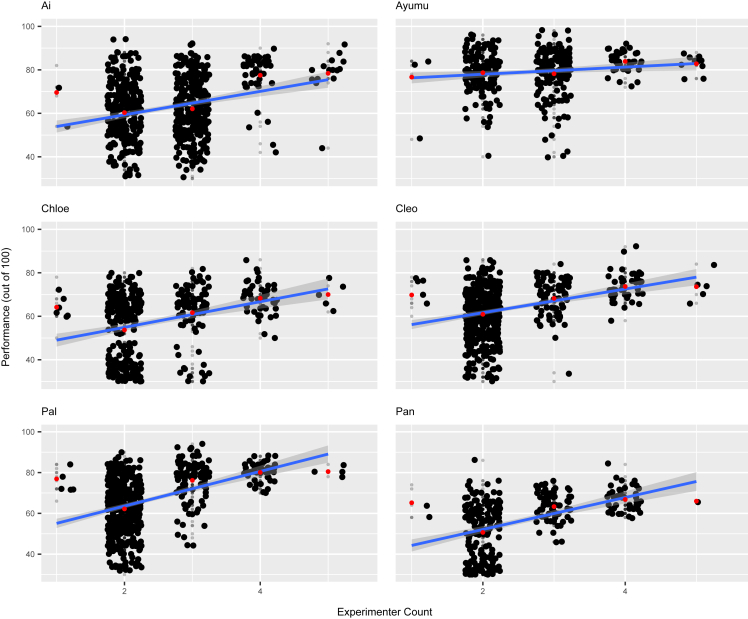
Chimpanzee performance at different experimenter audience counts for task type 3 (most difficult task) Scatterplots of performance out of 100% on task type 3 against experimenter counts. Plots are separated by individual, and subject names are listed in the top left corner of each plot. Included on each plot is an estimated linear model represented by a blue line, with the gray area representing a 95% confidence interval of the linear prediction. Red points represent the average performance for the corresponding experimenter count of that column. For the sake of clarity, horizontal and vertical jitter were applied to the data points. Each small gray point represents the performance for one experimental session. Larger black points represent exact performance values that were reached in more than one session. Sample sizes per individual were as follows, in order from left to right, top to bottom: *n* = 609, 332, 494, 515, 565, and 388. See also Table S8.

To more closely examine the difference between task types, we again conducted separate analyses for each task type. For task type 1, the fixed effects of experimenter and familiar audience count were both statistically significant (d.f. = 5, χ^2^ = 29.658, *p* < 0.001; d.f. = 5, χ^2^ = 5.905, *p* = 0.015; Table S8, respectively). The model estimated a 6% to 11% decrease in task type 1 performance per increase in experimenter count (Figure 2), and a 1% to 10% decrease for familiar audience count (experimenter 95% CI odds ratio: 0.890, 0.946; familiar 95% CI odds ratio: 0.907, 0.989; Table S8). The likelihood-ratio test for task type 2 indicated that familiar audience count was a significant fixed effect for this task type (d.f. = 5, χ^2^ = 9.540, *p* = 0.002). The relationship between familiar audience count and performance was positive for task type 2, with performance slightly increasing as familiar audience count increased (95% CI odds ratio: 1.011, 1.052; Table S8). In the model for task type 3, the fixed effect of experimenter count was statistically significant (d.f. = 5, χ^2^ = 14.600, *p* < 0.001), with performance increasing as experimenter count increased (95% CI odds ratio: 1.018, 1.057; Figure 3; Table S8). For more information on model 3, refer to Table S8.

## Discussion

To investigate whether chimpanzee task performance is influenced by the presence of audience members, we analyzed cognitive task data for multiple chimpanzees across various types of tasks. Our results suggest that their performance was affected by the presence of certain types of individuals and that this effect on performance also differed based on the type of task being performed. The chimpanzees’ performance in task type 1 was significantly affected by the number of experimenters and familiar audience members, with most chimps experiencing a decrease in performance as the experimenter and familiar audience count increased (Figure 2; Table S8). For task type 2, performance increased as familiar audience count rose, while for task type 3, experimenter count was found to have a significant positive relationship with task performance, the opposite of the trend for task type 1 (Figure 3; Table S8). There was no significant difference in performance for any task type as unfamiliar audience count increased, however (Table S8). Considering that chimpanzees can distinguish between familiar and unfamiliar people, it seems natural that the chimpanzees might care more about the judgment of familiar people, with whom they interact with in their daily life, compared to that of unfamiliar humans.

The results of this study indicate some possible complex audience effects on chimpanzees’ cognitive performance according to the difficulty of the tasks they worked on. The three task types differed in their cognitive load: task type 1 required knowledge of Arabic numeral order in an adjacent chain, task type 2 required knowledge of numeral order in a non-adjacent chain, and task type 3 was a memory task version of task type 2. Thus, task type 1 was theoretically the easiest task, and task type 3 was the most difficult one. This assessment was empirically confirmed by the chimpanzees’ actual performance data (Table S5). Here, we propose three alternative hypotheses to explain the divergent effects of audience on their cognitive performance across the tasks of varying difficulty. These hypotheses are founded on the basis of audience presence influencing different factors: reward value, cognitive load, and concentration.

Our first hypothesis explains the results from the viewpoint of a possible audience effect on motivation to obtain rewards in the presence of audience. The increase in performance for the difficult task type 3 as experimenter count increased could have been due to an increase in arousal that led to higher skill and awareness of the potential rewards for the task. A previous study on social facilitation effects in humans for a motor task that granted rewards based on performance found that higher pressure led to increased motivation toward the task, possibly due to a neurobiological mechanism causing the reward for performing the task to be perceived at a higher value while under stress.^37^ Because task type 3 was more difficult, more effort would have had to be exerted by the chimpanzees to receive their food rewards during this task. This could have caused the chimpanzees to perceive food rewards from task type 3 as higher value than from an easier task due to the difficulty in acquiring them, despite the fact that food rewards were consistent across task types. Thus, the increase in experimenter audience counts could have led to an increase in pressure to perform well for the chimpanzees under task type 3, increasing their motivation to obtain food rewards from this task via the mechanism described by Chib et al.,^37^ and thus improving their performance as this pressure increased. In fact, this mechanism could have occurred despite the chimpanzee’s general knowledge of what kind of food rewards they would obtain, as their perception of the food’s value would affect their motivation and performance regardless of the reward’s value. Conversely, because acquiring food rewards from task type 1 was comparatively easier, this mechanism may not have occurred for this task, and as a result, the chimpanzees were simply more distracted under this task type and so their performance suffered as audiences increased.

Our second hypothesis approaches the results from the perspective of an audience effect on cognitive load.^38^ The increase in performance for task type 3 could be due to a higher cognitive load imposed on the chimpanzees by the presence of familiar audiences causing an improvement in their skills. Roberts & Roberts found that gesturing in chimpanzees can surprisingly become more complex under socially stressful conditions, and they suggest that how chimpanzees interact and manage their social relationships can be affected by stress levels and the dominance rank of those nearby.^39^ In fact, the authors also argue that while the presence of audience members can be a stressor to chimpanzees, this stress can motivate them to modify their behavior more carefully; for example, during mating, chimpanzees were found to change their gestures based on whether a dominant male was watching them or not, a situation which can be stressful for lower-ranking individuals despite familiarity with their observers.^26^^,^^40^ Although it is difficult to determine the dominance relationships between the chimpanzees at PRI and the familiar audience members and experimenters, their mere presence may have been a similar stressor for the chimpanzees, and this may have in turn caused them to perform even better than usual. It may be the case that chimpanzees demonstrate an increase in ability under stress not just for gesturing and communication, but for cognitive tasks such as this one as well. The opposite trend in performance for task type 1 could therefore be due to the difference in difficulty again; similar to how right and left-handed gestures are used for situations of varying complexity,^26^ the different task types might require different skillsets that are variably affected by the presence of audience members in chimpanzees.

Our third explanation is based on the distraction hypothesis, in which there is a potential audience effect on concentration. In the distraction hypothesis, performance for more rigid and rule-based tasks is expected to decrease under pressure, while performance for more flexible tasks requiring deeper thought is expected to increase.^41^^,^^42^^,^^43^ This is because situations with increased pressure can cause learned skills to be more difficult to remember.^43^^,^^44^ Because task type 1 only required the knowledge of the order of numbers, the method of solving tasks of this type would always be fixed. On the other hand, task type 3 was more variable in that it required not only the knowledge but also memorization of the new set of numbers and their positions for each trial. This higher level of involvement required for task type 3 could have led to an increase in the performance for this task while under the pressure of increasing audience size, based on the distraction hypothesis. The decrease in performance for task type 1 would also coincide with this hypothesis.

For task type 2, there was a statistically significant positive relationship between familiar audience count and performance, but not for experimenter count (Table S8). Although task types 1 and 2 shared similar differences in performance with task type 3 (Table S5), it is possible that some element in the design of task type 2 causes the chimps to be less affected in performance while performing the task in the presence of familiar people and experimenters. Task type 1 always involved adjacent numbers, whereas in task type 2, the numbers that appeared were never adjacent to each other. While the two task types appear to be similar in difficulty based on the chimpanzees’ performance (Table S5), we might still expect task type 2 to be more difficult, due to the numbers being non-adjacent. It is unclear why there is a lack of a clear trend in performance for task type 2, but one potential explanation is that task type 2 was difficult enough to warrant less attention toward audience members, but not quite as challenging as task type 3, and thus the chimpanzees’ performance was not as enhanced by the presence of more experimenters. Further investigation on the design of these task types could shed more light on these results.

Overall, there are several potential explanations for the chimpanzees’ trends in performance across task types and audience sizes. In our future studies, we hope to more explicitly examine the possible mechanisms behind these audience effects in chimpanzees through modifying the experimental design to test for specific phenomena, such as the distraction hypothesis. Additionally, we also aim to test the effect of varying conspecific audience sizes, as the potential differences in how the chimpanzees view human versus chimpanzee audiences and how they might impact their performance are also of interest. Although with our present results it is difficult to draw conclusions regarding reputation management and whether the chimpanzees may have given special care toward how their audiences perceived their performance, in future studies, we also aim to incorporate this aspect into our experimental design, to examine potential similarities and differences to how chimpanzees and humans manage their reputation while performing difficult tasks in the presence of others. Such research will help us further examine what type of social pressures can affect the behavior of chimpanzees and reveal how these aspects relate to differences in ape and human communication and sociality.

Finally, we would like to emphasize the unique situation of our chimpanzee participants at PRI; many of them have grown with and enjoyed the company of the experimenters, familiar audience members, and other people for much of their lives, and thus may have special affiliative relationships with humans that were uniquely reflected in these results and would not be found in other chimpanzee populations, especially those in the wild. There is still much to uncover about the evolutionary path that has led humanity to where we are today, and through our special relationship shared with chimpanzees, both as evolutionary cousins and, in the case of unique locations such as PRI, as friends and colleagues, we can continue to learn more about ourselves and our shared ancestry.

### Limitations of the study

To our knowledge, this is one of the first studies investigating the presence of audience effects in non-human animals with a set of uniquely large and long-term data, though we acknowledge there are several limitations to our study that we wish to address. In the future, we need to investigate the effects of conspecific audience members, since this study only examined the effects of human witnesses. It may be the case that chimpanzees pay more care toward the judgment of fellow chimpanzees, or perhaps are sometimes too focused on food rewards to care for the presence of humans. Additionally, in our present study, we were unable to introduce a proper control condition under which no humans or experimenters were present, since human presence was always required to manage the touch screen tasks. Future studies involving conspecifics should introduce a control condition with no conspecifics present and could explore the possibility of remotely controlling the touch screen and feeder systems to obtain data with no other people or chimpanzees present. Additionally, while it was possible to estimate the difference in difficulty between the cognitive tasks used in this study, there was no empirical method to quantify the varying cognitive demands of the tasks other than through the task design and the chimpanzees’ performance post-task. Thus, in future studies, we also aim to design the cognitive tasks in such a way that difficulty can be included as an objective measure in our analysis. In addition to this, although additional rewards were not provided based on difficulty or task type during this study, it would have been preferable to fix the type of food reward across all conditions, to avoid preferences for certain food types among the chimpanzees affecting their performance. However, the chimpanzees were given a variety of food rewards over the many years of data collection, and given that specific food types were never intentionally provided for specific task types, we expect that such biases are unlikely to have appeared in our data.

## Resource availability

### Lead contact

Further information and requests for resources should be directed to and will be fulfilled by the lead contact, Christen Lin (linchristen@gmail.com).

### Materials availability

This study did not generate new unique reagents.

### Data and code availability

• All code and data have been deposited at Mendeley Data and are publicly available. DOI is listed in the key resources table.
• This paper does not report original code.
• Any additional information required to reanalyze the data reported in this paper is available from the lead contact upon request.

## Acknowledgments

We thank all the chimpanzee participants for their continuous collaboration in this study. We would like to give special thanks to Dr. Tetsuro Matsuzawa, Dr. Ikuma Adachi, Dr. Misato Hayashi, Ms. Mari Hirosawa, Ms. Tomoko Takashima, Ms. Etsuko Ichino, Ms. Akemi Hirakuri, and other PRI staff, including care takers and veterinarians, for their arrangement of and support with the chimpanzee experiments at PRI (recently re-organized into the Center for the Evolutionary Origins of Human Behavior: EHUB), Kyoto University, and Dr. James Brooks for their support with the statistical analyses. This study was financially supported by 10.13039/501100001691MEXT/JSPS KAKENHI (16002001, 20002001, 24000001, and 16H06283 to Tetsuro Matsuzawa, 14J00488 to A.M., and 19H00629, 22H04451, and 24H02200 to S.Y.) and JST FOREST program (JPMJFR221I to S.Y.). This work was supported by the Cooperative Research Program of the Primate Research Institute, 10.13039/501100005683Kyoto University (2020-C-10 and 2021-C-2).

## Author contributions

Conceptualization, S.Y.; investigation, A.M.; formal analysis, C.L., A.M., and S.Y.; writing – original draft, C.L.; writing – review and editing, A.M. and S.Y.; supervision, S.Y.; funding acquisition, S.Y.

## Declaration of interests

The authors declare no competing interests.

## STAR★Methods

### Key resources table

**Table:** 

REAGENT or RESOURCE	SOURCE	IDENTIFIER
**Deposited data**		

	This paper	Mendeley Data: https://doi.org/10.17632/494hd7pksd.2
	Akiho Muramatsu & Tetsuro Matsuzawa	https://doi.org/10.3390/ani13050774

**Experimental models: Organisms/strains**		

Chimpanzees (*Pan troglodyte*)	Primate Research Institute (PRI, recently re-organized as the Center for the Evolutionary Origins of Human Behavior: EHUB)	https://shigen.nig.ac.jp/gain/

**Software and algorithms**		

R	R Foundation	https://www.r-project.org/

### Experimental model and study participant details

Participants were six chimpanzees (three juveniles and their mothers) living with their group mates in an enriched environment at the Primate Research Institute (PRI, recently re-organized as the Center for the Evolutionary Origins of Human Behavior: EHUB) of Kyoto University located in Inuyama, Japan.

#### Chimpanzee participants

Refer to Table S1 for further details.

##### Ai

• Species: Pan troglodyte
• Sex: Female
• Age (at the start of the study): approximately 33 years

##### Chloe

• Species: Pan troglodyte
• Sex: Female
• Age (at the start of the study): 28 years

##### Pan

• Species: Pan troglodyte
• Sex: Female
• Age (at the start of the study): 25 years

##### Ayumu

• Species: Pan troglodyte
• Sex: Male
• Age (at the start of the study): 9 years

##### Cleo

• Species: Pan troglodyte
• Sex: Female
• Age (at the start of the study): 9 years

##### Pal

• Species: Pan troglodyte
• Sex: Female
• Age (at the start of the study): 8 years

The chimpanzees in PRI were cared for based on the Guideline of Care and Use of Nonhuman Primates, KUPRI, and the Japanese Act on the Welfare and Management of Animals. The chimpanzees, including our participants, were regularly fed three times per day, and additional food was given several times per day for enrichment purposes. The amount of food they consumed daily was controlled for each individual to maintain their health. Chimpanzees were selected for this experiment based on their performance on previous touch screen experiments. For the individuals who participated in the cognitive experiments, the amount they ate during the experiment was subtracted from their regular meal for that day. In other words, our participants would receive the same amount of food per day whether they participated in the experiment or not, and whether they performed well or poorly on the tasks. They could access water freely in outdoor enclosures and rooms.

The data collection period started in July 2009 and ended in January 2016. At the beginning of data collection, the three juveniles were around 9 years old, and their mothers were 25–33 years old (refer to Table S1 for more details). Data collection was done as a part of a larger project studying chimpanzee cognition at PRI. The chimpanzees were free to come and go as they pleased, and spent the time between experimental sessions in an enriched outdoor enclosure where a group of 14 individuals lived (one chimpanzee passed away during the study period). The experimenters would call the name of a particular chimpanzee to invite them into the cognitive testing touch screen booth, and the chimpanzees were free to choose whether or not they wanted to accept this invitation to partake in the cognitive tests. Thus, the data used in this study was recorded only on days during which the chimpanzees were willing to enter the experimental booth to participate in the experiments. The data collection process followed the Guideline of Care and Use of Nonhuman Primates, KUPRI, and was approved by the Animal Welfare and Care Committee of PRI. It also followed the Japanese Act on the Welfare and Management of Animals.

### Method details

Data from six chimpanzees living at PRI was taken over the course of six years from 2009 to 2015. We analyzed the chimpanzees’ performance on three different types of cognitive tasks with multiple variants within each type of task. These tasks were developed primarily for investigating chimpanzees’ number concept and working memory^34^^,^^35^^,^^36^^,^^45^^,^^46^ as a part of the Ai project since 1978.^47^^,^^48^ The data used in the current study were obtained within this line of studies, and some parts of these data were already published in another paper reporting chimpanzees’ learning of sequence order in the Range 1 to 19.^49^ This study re-analyzes the data for the completely different research purpose of investigating audience effects in chimpanzees.

#### Apparatus

Each touch by the participant in each cognitive computer task was detected by a CRT monitor with a touch screen (Mitsubishi Electric Engineering 15-inch LCD touch screen monitor: TSD-FT157-MN and TSD-AT1515-MN). The monitor was encased in a translucent acrylic box. For an adult chimpanzee sitting in front of the monitor, the center of the monitor was placed at eye level. When testing a mother and child at the same time, experimenters would use a “twin booth” consisting of two identical booths located side by side (Figure A). Each booth was 1.8 m by 1.8 m by 2 m in height. For most mother-child pairs, mothers came to the booth on the far side, while the children chose the side closer to the entrance. During each experimental session, chimpanzees could see the experimenters and visitors through the transparent acrylic panels of the experimental booth.

The chimpanzees participated daily in several types of cognitive tasks on a fixed schedule from Mondays to Fridays (Table S3). The computer task was controlled by standard PC-type computers running either Windows XP or Windows 7 OS. The program which controlled the cognitive computer tasks was made in Visual Basic version 6.0.^50^ The food reward was delivered by an automatic feeder (Bio Medica: BUF-310-P50) which connected to the computer. Thus, daily cognitive test sessions were fully automated with the computer programs to avoid interference caused by experimenters, while the experimenters interacted with the chimpanzees and gave them some additional food and/or verbal encouragement during the intervals between sessions.

#### Data collection & procedure

Each trial of the cognitive computer task went as follows: a circle would first appear at the bottom of the monitor as a start key. Once the participant touched the start key with their finger, the circle would disappear, and Arabic numeral stimuli appeared on the monitor. The correct response was followed by a chime and automated food delivery. The food reward was a piece of apple (5–8 mm cubes, a whole apple was cut into about 200–300 pieces) or a full- or half-size small raisin. The chimpanzees ate apples as part of their daily regular meal, and the apples used as experimental rewards were taken from this daily meal for each individual. Food rewards were randomly distributed, and were not given based on the type of task being performed. An incorrect response was followed by a buzzer sound and a 3-s blackout. In general, each session consisted of 50–90 trials and was completed in 3–5 min. Experimenters conducted 5 to 10 sessions per day for each participant.

The participants’ performance was automatically recorded as electronic data. Experimenters also recorded their performance and the number of experimenters and visitors manually. Experimenters were considered to be those involved in managing the touch screen systems and the automatic feeders, as well as re-filling the feeders during the experiments. Familiar audience members were those the chimpanzees were familiar with, but were not actively involved in the experiment beyond simply observing. Unfamiliar audiences were also observers, but were not familiar to the chimpanzees. We used the data of three types of computer cognitive tasks in this study, referred to as Task Types 1, 2, and 3. These tasks used Arabic numerals as stimuli, requiring participants to touch the numerals in ascending order. In Task Type 1, numerals from a range of 1–19 would always appear sequentially on the screen, but the total number of numerals that would appear in one session varied.^34^^,^^49^ Thus, Task Type 1 required recognition of the numerals and memorization of their correct order. For Task Type 2, nonadjacent numerals appeared on the screen, but the number of stimuli was fixed in each session.^34^^,^^51^^,^^52^ In Task Type 3, the conditions were the same as for Task Type 2, but with one key difference: once the initial numeral was touched, the other numerals were masked with checkered pattern rectangles.^34^^,^^35^^,^^36^ Thus, for this particular task participants had to memorize the location and order of the numeral stimuli in order to touch them in ascending order, and so Task Type 3 was generally the most cognitively demanding task among the three. Upon completion of one set of numerals of a given task type, the chimpanzees were rewarded automatically with 1 unit of food, depending on the food reward being used that day. The amount of food reward did not differ with task type or difficulty, and the type of food reward was not associated with any specific task types either. While one of these three task types was noted for each session, a more specific task ID was also recorded, which contained the specific parameters of each session, such as the specific range of numbers used, which influenced the task difficulty. Thus, there was variation even within tasks of the same type, due to the potential difference in the quantity of numeric stimuli between different sessions (see supplementary data for detailed experimental schedule). Further variations of numerical tasks were also performed by the chimpanzees, but due to low sample size we excluded them from this current study.

### Quantification and statistical analysis

For the analyses in this study, we used data of 2182 sessions across 760 days for Task Type 1, 4134 sessions across 794 days for Task Type 2, and 2903 sessions across 792 days for Task Type 3. Overall, data collection spanned over the course of 6 years. Sessions involving nine or more total audience members were excluded from data analysis due to small sample size (accounting for less than 3% of the total data), as well as trials for certain task ID’s, since due to their high specificity these tasks lacked repeated trials. For our analysis, we created multiple groups of models incorporating random intercepts, with each focusing on certain variables of interest to assess their effects on task performance. For all models, the task serial number variable was normalized before analysis to avoid modeling errors. Additionally, a likelihood-ratio test was performed for each model to further evaluate the variable of interest (likelihood-ratio tests were performed against each corresponding null model). 95% confidence intervals were also constructed for the fixed effect parameters of each GLMM. We also graphically summarized the mean values for variables of interest. All analysis was conducted in RStudio 4.1.0, and all GLMM’s were of the binomial family and performed with the “glmer” function from the lme4 package.^53^ Likelihood-ratio tests were performed with the “drop1” function with the chi-squared test, and ANOVA analysis and Tukey’s HSD were performed with the “aov” and “TuekyHSD” functions, respectively, from R.^54^ 95% confidence intervals were constructed with the “confint” function, using the Wald method (see Table S2 for detailed explanation of all variables used in our analysis).
