# Supplementary material for: Audience presence influences cognitive task performance in chimpanzees
Source: iScience. 2024 Nov 8;27(11):111191. doi: 10.1016/j.isci.2024.111191 (PMC11607530; doi:10.1016/j.isci.2024.111191)
Supplement: Document S1. Figure S1 and Tables S1–S8 [file mmc1.pdf]

iScience, Volume *27*

Supplemental information

Audience presence influences cognitive task performance in chimpanzees

Christen Lin, Akiho Muramatsu, and Shinya Yamamoto

Supplemental Items

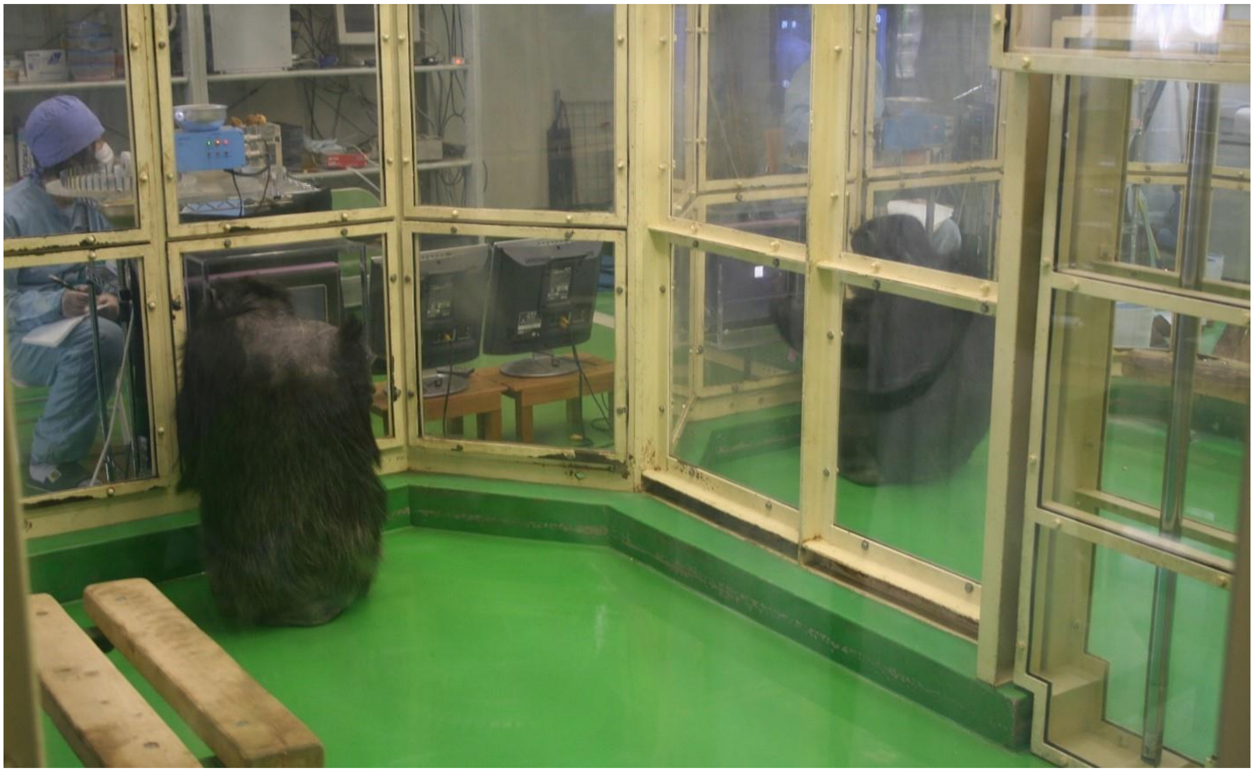

Figure S1. Image of the twin booth used to test two chimpanzees at the same time, Related to STAR Methods. Chimpanzees were tested in a booth containing acrylic transparent panels, allowing chimpanzee participants and human audience members to see each other during experiments. Touch screens were attached to one of the acrylic panels, and food rewards were dispensed through a hole on the side of these panels.

Name	Sex	GAIN ID	Mother	Rearing history	Date of birth	Age
Ai	F	0434	NA	Wild-born	1976?	33 y
Chloe	F	0441	Charlotte	Nursery-peer	1980, Dec 12	28 y, 7 m
Pan	F	0440	Puchi	Nursery-peer	1983, Dec 7	25 y, 7 m
Ayumu	M	0608	Ai	Mother	2000, Apr 24	9 y, 3 m
Cleo	F	0609	Chloe	Mother	2000, Jun 19	9 y, 1 m
Pal	F	0611	Pan	Mother	2000, Aug 9	8 y, 11 m

Table S1. The six chimpanzee participants for this study, Related to STAR Methods. Information on chimpanzee participants' gender and rearing history. Age is at the beginning of the present study. In the rearing history column, 'Mother' indicates that the individual was reared in captivity by their biological mothers, 'Nursery-peer' indicates that the individual was reared by human caregivers and conspecific peers, and "Wild-born" indicates that the individual was born in the wild and taken into captivity. For further information please visit GAIN (https://shigen.nig.ac.jp/gain/; Great Ape Information Network; the online studbook of Japanese apes) and type their names in the search bar.

Variable name	Description
Response variable:	
Accuracy	Proportion of correct answers, calculated for each experimental session.
Fixed effects:	
Total audience count	The total number of human audience members present for a given session, including familiar and unfamiliar individuals, as well as number of experimenters present.
Task serial number	The *n*th number time that the given participant has performed a given task type.
Familiar audience count	The number of familiar human audience members present. Audience members were considered familiar if the chimpanzees had seen them previously in the experimental area.
Unfamiliar audience count	The number of unfamiliar human audience members present. Audience members were considered unfamiliar if the chimpanzees had not seen them before.
Experimenter count	The number of people managing the experiment present (e.g. setting up the touchscreen devices, managing the automatic feeders, reward the chimpanzees with food between sessions, etc.)
Task Type	All tasks utilized in this study could be categorized into one of 3 different general task types: Mask, VarNumMix, and Nonmask.
Random effects:	
Subject	The chimpanzee participant for any given trial. Subject was included as a random effect in some models to account for potential variation in individual skill differences in performing the various tasks.
Task ID	The specific type of task performed during a given experimental session. While each Task ID could be categorized under one of the three task types, they differed in their specific parameters and therefore difficulty as well, even within broad task types. Task ID was included as a random effect to account for potential variation in task difficulty.

Table S2. Detailed explanation of all the variables used in our analysis, Related to STAR Methods. Table containing in-depth explanations of all the variables used for constructing the GLMMs used for analysis.

	Mother		Child	
Morning 1<br>(9:00-10:00)	Ai	Task Type 1<br>Task Type 2<br>Task Type 3<br>(Other cognitive task)	Ayumu	Task Type 1<br>Task Type 2<br>Task Type 3<br>(Other cognitive task)
Morning 2<br>(10:00-11:00)	Chloe	Task Type 1<br>Task Type 2<br>Task Type 3<br>(Other cognitive task)	Cleo	Task Type 1<br>Task Type 2<br>Task Type 3<br>(Other cognitive task)
Afternoon<br>(13:30-14:30)	Pan	Task Type 1<br>Task Type 2<br>Task Type 3<br>(Other cognitive task)	Pal	Task Type 1<br>Task Type 2<br>Task Type 3<br>(Other cognitive task)

Table S3. Experimental schedule and task types of six chimpanzees, Related to STAR Methods. This table lists the daily schedule for each of the chimpanzee participants during this study. Other cognitive tasks refer to further variations of numerical tasks that were performed but were excluded from analysis due to low sample size.

One-Way ANOVA of Performance vs. Task Type

	d.f.	sum sq.	mean sq.	F value	P value
Task Type	2	569267	284634	1420	**< 0.001**
Residuals	9216	1847049	200	-	-

Table S4. Results of a one-way ANOVA comparing performance (accuracy) and the 3 task types, Related to STAR Methods. This Table details the results of ANOVA aiming to compare differences in the chimpanzees' accuracy between the different task types.

Tukey multiple comparison of means

pair	mean diff	lower	upper	P value
Task Type 1 vs. Task Type 2	-0.135	-1.013	0.743	0.931
Task Type 1 vs. Task Type 3	-17.006	-17.946	-16.066	**< 0.001**
Task Type 2 vs. Task Type 3	-16.871	-17.675	-16.068	**< 0.001**

Table S5. Results of Tukey HSD performed on ANOVA from Table S2, Related to STAR Methods. Lists comparisons between every combination of the 3 task types are listed with the mean differences and their corresponding 95% confidence interval values, along with respective P values.

Model 1: Does the presence of extra people affect performance?

Model	Likelihood Ratio Test Results			95% Confidence Interval (fixed effects)			
	d.f.	chi-sq.	P value	Lower CI	Upper CI	Lower CI (odds ratio)	Upper CI (odds ratio)
**Interaction between Presence and Task Type**							
Task serial number	4	1468.700	< 0.001				
Presence*Task type	9	9.870	**0.007**				
**Presence of audience (Task Type 1)**							
Intercept				1.088	2.046	2.968	7.735
Presence of audience (fixed effect)	5	2.523	0.112	-0.166	0.018	0.847	1.018
**Presence of audience (Task Type 2)**							
Intercept				0.691	2.141	1.995	8.508
Presence of audience (fixed effect)	5	0.010	0.917	-0.041	0.037	0.960	1.038
**Presence of audience (Task Type 3)**							
Intercept				-0.489	1.443	0.613	4.233
Presence of audience (fixed effect)	5	0.920	0.338	-0.060	0.021	0.941	1.021

Table S6. Summary of all Model 1 GLMM results, Related to STAR Methods. The first model was made incorporating all 3 tasks, and separate analysis for each task type was conducted to further examine differences between the 3 task types. In the base model, the fixed effects were the interaction effect between the presence of extra audience members (which excluded experimenters) and task type, as well as task serial number. The presence variable was coded as “No” for none present and “Yes” for one or more audience members present. The model formula for the interaction between presence and task type was as follows: performance ~ presence of audience members*task type + task serial number + random effects. For the 3 follow-up models, only data from each task type was used in each model. The formulas for these were: performance ~ presence of audience members + task serial number + random effects. The random effects were always as follows: (1|subject) + (1|TaskID). Listed are the results of the LRT for each model against its corresponding null model, performed using the chi-squared test. The 95% confidence intervals, along with the corresponding converted odds ratio values for each fixed effect parameter, are listed as well.

Model 2: Does the number of people present affect performance?

Model	Likelihood Ratio Test Results			95% Confidence Interval (fixed effects)			
	d.f.	chi-sq.	P value	Lower CI	Upper CI	Lower CI (odds ratio)	Upper CI (odds ratio)
**Type**							
Task serial number	4	1391.010	< 0.001				
Total audience count*Task type	9	40.170	**< 0.001**				
**Total audience count (Task Type 1)**							
Intercept				1.199	2.188	3.316	8.919
Total audience count (fixed effect)	5	27.931	**< 0.001**	-0.064	-0.029	0.938	0.971
**Total audience count (Task Type 2)**							
Intercept				0.693	2.145	2.000	8.538
Total audience count (fixed effect)	5	0.040	0.846	-0.010	0.008	0.990	1.008
**Total audience count (Task Type 3)**							
Intercept				-0.502	1.423	0.605	4.150
Total audience count (fixed effect)	5	0.840	0.358	-0.005	0.014	0.995	1.014

Table S7. Summary of all Model 2 GLMM results, Related to Figure 1. The initial model was made incorporating all 3 tasks, and separate analysis for each task type was performed to further examine differences between the 3 task types. In the first model, the fixed effects were the interaction between total audience count (which was the sum of the number of experimenters, familiar audience, and unfamiliar audience present) and task type, as well as task serial number. The first model formula was as follows: performance ~ total audience count*task type + task serial number + random effects. The follow-up models using data from each task type separately used the following formula: performance ~ total audience count + task serial number + random effects. The random effects were always as follows: (1|subject) + (1|TaskID). Listed are the results of the LRT for each model against its corresponding null model, performed using the chi-squared test. The 95% confidence intervals, along with the corresponding converted odds ratio values for each fixed effect parameter, are listed as well.

Model 3: Does the specific type of audience affect performance?

Model	Likelihood Ratio Test Results			95% Confidence Interval (fixed effects)			
	d.f.	chi-sq.	P value	Lower CI	Upper CI	Lower CI (odds ratio)	Upper CI (odds ratio)
Interaction between All Audience Types and Task Type							
Task serial number	4	1192.120	< 0.001				
Experimenter count*Task type	9	45.270	< 0.001				
Familiar count*Task type	9	30.920	< 0.001				
Unfamamiliar count*Task type	9	0.290	0.318				
Experimenter and Familiar audience count (Task Type 1)							
Intercept				1.332	2.212	3.789	9.136
Experimenter count (fixed effect)	5	29.658	< 0.001	-0.117	-0.055	0.890	0.946
Familiar audience count (fixed effect)	5	5.905	0.015	-0.098	-0.011	0.907	0.989
Experimenter and Familiar audience count (Task Type 2)							
Intercept				0.756	2.042	2.129	7.708
Experimenter count (fixed effect)	5	0.030	0.861	-0.015	0.018	0.985	1.018
Familiar audience count (fixed effect)	5	9.540	0.002	0.011	0.050	1.011	1.052
Experimenter and Familiar audience count (Task Type 3)							
Intercept				-0.408	1.175	0.665	3.237
Experimenter count (fixed effect)	5	14.600	< 0.001	0.018	0.056	1.018	1.057
Familiar audience count (fixed effect)	5	1.690	0.193	-0.035	0.007	0.966	1.007

Table S8. Summary of all Model 3 GLMM results, Related to Figures 2 & 3. The initial model was made incorporating all 3 tasks, and separate analysis for each task type was performed to further examine differences between the 3 task types. In this model, the fixed effects were the interaction between experimenter count and task type, the interaction between familiar audience count and task type, the interaction between unfamiliar audience and task type, and task serial number. The initial model formula was as follows: performance ~ experimenter count*task type + familiar audience count*task type + unfamiliar audience count*task type + task serial number + random effects. The follow-up models used data from each task type separately, and only included experimenter and familiar audience counts as fixed effects. The formula for these was as follows: performance ~ experimenter count + familiar audience count + task serial number + random effects. The random effects were always as follows: (1|subject) + (1|TaskID). Listed are the results of the LRT for each model against its corresponding null model, performed using the chi-squared test. The 95% confidence intervals, along with the corresponding converted odds ratio values for each fixed effect parameter, are listed as well, constructed using the Wald method.
